# The Impact of Mineral Supplementation on Polycystic Ovarian Syndrome

**DOI:** 10.3390/metabo12040338

**Published:** 2022-04-08

**Authors:** Tahra ElObeid, Marwa Osman Awad, Vijay Ganji, Joyce Moawad

**Affiliations:** 1 Human Nutrition Department, College of Health Sciences, QU Health, Qatar University, Doha P.O. Box 2713, Qatar; vganji@qu.edu.qa (V.G.); jmoawad@qu.edu.qa (J.M.); 2 Department of Northern Region Operations, Primary Health Care Corporation, Doha P.O. Box 26555, Qatar; moawad@phcc.gov.qa

**Keywords:** PCOS, polycystic ovarian syndrome, mineral supplementation, magnesium, zinc, selenium, chromium

## Abstract

Polycystic ovary syndrome (PCOS) is an endocrinopathy that is common among women of reproductive age. It is a heterogeneous disorder with an unknown etiology. Different strategies have been proposed for the treatment of PCOS. Recent studies recommend supplementation with specific minerals for treating various PCOS phenotypes. We searched PubMed, Google Scholar, and SCOPUS databases by using search terms combining PCOS with the supplementation of magnesium, zinc, selenium, or chromium. This review presents a narrative concerning the association between PCOS and magnesium, zinc, selenium, and chromium supplementation. We review findings from various randomized controlled trials and meta-analyses conducted in women of childbearing age with PCOS. Recent reports highlight the beneficial effect of minerals on the clinical and metabolic symptoms of PCOS. Further studies are required to establish the appropriate dosage level of these minerals for ameliorating the pathologies associated with PCOS because of the potential health risks linked with higher doses.

## 1. Introduction

Polycystic ovary syndrome (PCOS) is a common heterogeneous endocrinopathy in women of reproductive age, affecting one in five women [1]. PCOS is an epigenetic disorder, and various environmental factors such as diet, physical activity, and other lifestyle factors can contribute to its pathology and prevalence [2]. Because the etiology of PCOS is not clearly understood, treating this ailment poses a challenge. It is manifested by a combination of anovulation, excess androgens (testosterone), dysfunction of the ovaries, and altered ovarian morphology (polycystic) [3]. These manifestations lead to the dysregulation of reproduction, leading to endocrine and metabolic abnormalities such as infertility, an irregular menstrual cycle [4], hyperinsulinemia, insulin resistance (IR), hyperglycemia, glucose intolerance, dyslipidemia, and obesity [5]. Coincidently, these biochemical derangements are also components of metabolic syndrome (MetSyn) [6]. Rotterdam criteria are widely used in the diagnosis of PCOS. A woman is diagnosed with PCOS if she presents two out of the three following criteria: hyperandrogenism, ovulatory dysfunction, and polycystic ovarian morphology [3].

Recent studies have reported a sub-optimal concentration of minerals such as magnesium, zinc, selenium, and chromium in women with PCOS [7,8,9]. Low concentrations of these minerals may be associated with the metabolic and clinical pathologies associated with PCOS. In fact, several of these minerals are components (cofactors) of enzymes or coenzymes that regulate various enzymatic reactions related to reproduction [10]. Therefore, many researchers have investigated the association between mineral supplementation and PCOS phenotypes. However, the results are contradictory and inconclusive. Therefore, to better understand the role of minerals in PCOS, we conducted a review to explore the relationship between the supplementation of magnesium, zinc, selenium, and chromium and the clinical and metabolic biomarkers of PCOS phenotypes.

## 2. Methods

This narrative review aims to address the effect of mineral supplementation on PCOS phenotypes. It is based on papers published between 2015 and December 2021 found in PubMed, Google Scholar, and SCOPUS. In our search, the following combination of key terms was used: “polycystic ovary syndrome and mineral supplementation”, “effects of mineral supplementation on polycystic ovary syndrome”, “polycystic ovary syndrome and magnesium supplements”, “polycystic ovary syndrome and selenium supplements”, “polycystic ovary syndrome and zinc supplements”, and “polycystic ovary syndrome and chromium supplements”. In this review, we focus on the findings from meta-analyses and randomized controlled trials (RCT) from 2015 to 2021. The reviewed studies are published in the English language and conducted in humans. The results are screened for relevance to the review topic. An additional manual search was conducted using the references listed in selected studies to detect other relevant trials.

## 3. Findings of Reviewed Papers between 2015–2021 and Discussion

### 3.1. Magnesium

Magnesium is an essential macromineral abundantly found in the human body and involved in more than 300 biochemical reactions that regulate carbohydrate, fat, and protein metabolism; DNA and RNA synthesis; neuro and muscular transduction; and blood pressure. It is the most important intracellular cation. The majority of the body’s magnesium is found in bones (about 60%), and the remaining is in soft tissues and in the extracellular environment [11]. Low serum magnesium (˂0.75 mmol/L) is more common in the Western population. Symptoms of hypomagnesemia are non-specific and may include nausea, vomiting, muscle weakness, fatigue, and anorexia. Severe magnesium deficiency may lead to an irregular heartbeat, tetanus-like symptoms (tremors, cramps, and spasms), and seizures [12].

The role of magnesium in endocrinological disorders is still emerging. Magnesium deficiency may result in several biochemical derangements associated with the gynecological pathologies of PCOS [13]. A study from India showed that women with PCOS (*n* = 60, 18–40 years old) had a significantly lower concentration of serum magnesium compared to age-matched controls. In these women, magnesium showed a significant inverse association with fasting blood glucose and serum lipids such as total cholesterol, triglycerides, and low-density-lipoprotein (LDL) cholesterol. A recent meta-analysis found a significant decrease in serum magnesium concentrations in patients with PCOS compared to non-PCOS controls (−0.09 mmol/L; *p* = 0.01). Additionally, overweight and obese patients [body mass index (BMI) ≥ 25 kg/m^2^] with PCOS had a lower concentration of magnesium compared to control subjects (−0.07 mmol/L; *p* = 0.02) [14].

Recent findings suggest that magnesium may improve the clinical symptoms of PCOS either through its role in improving glucose homeostasis [15] and reducing androgens [16] or due to its anti-inflammatory and antioxidant effects [17]. A study conducted on Chinese women with PCOS (*n* = 1000) suggested that women with high magnesium concentrations (4th quartile) had significantly lower fasting blood glucose, HOMA-IR, and testosterone [18]. An observational cohort study conducted in women of childbearing age with PCOS (*n* = 137) showed a negative association between magnesium intake and IR, C-reactive protein, and testosterone but a direct association with high-density-lipoprotein cholesterol [15]. A systematic review of epidemiological studies by the authors of [19] found a direct association between serum magnesium and IR in women with PCOS. On the other hand, based on the review of RCT, those same authors did not find a consistent impact of magnesium supplementation on IR.

A recent study reported a significant hormonal effect of magnesium supplementation in women with PCOS [16]. Farsinejad-Marj et al. [16] conducted a study on 60 women (20–45 years old) with PCOS. Following the consumption of 250 mg of magnesium supplements for 8 weeks, several clinical and metabolic improvements in pathologies of PCOS were reported. There were decreases in BMI and serum testosterone concentrations, as well as an increase in the concentrations of serum dehydroepiandrogens (DHEA) and luteinizing hormone. However, these investigators found no effect of magnesium supplementation on glycemic control or serum lipids in women with PCOS.

Evidence suggests a synergistic effect of combination of magnesium with other nutrients in improving pathologies associated with PCOS. A randomized, double-blind, placebo-controlled trial performed in women with PCOS (*n* = 60; 18–40 years old) showed a synergistic impact of magnesium and vitamin E supplementation on the clinical and metabolic symptoms of PCOS following daily supplementation with 250 mg and 400 mg, respectively [20]. In this study, a significant decrease in the Ferriman-Gallwey score (a scale that is commonly used to measure the severity of hirsutism) (14.9 vs. 14.6; β = −0.37) and in the serum high-sensitivity C-reactive protein (hs-CRP) (3.7 mg/L vs. 3.1 mg/L; β = −0.67) and a significant increase in total antioxidant capacity (TAC) (522 mmol/L vs. 590 mmol/L; β = 66.3) were reported [20]. These beneficial effects are likely due to the antioxidant and anti-inflammatory properties of magnesium and vitamin E.

Additionally, the effect of the co-supplementation of magnesium (250 mg of magnesium oxide) and zinc (220 mg of zinc sulfate) for 12 weeks on the metabolic features of PCOS was evaluated in a randomized, double-blind, placebo-controlled study [17]. This study was conducted on 60 women with PCOS aged 18–40 years old. These findings showed a significant decrease in hs-CRP concentrations, a significant increase in plasma TAC, and downregulation of the gene expression of interleukin-1 and tumor necrosis factor-α) [17].

The exact molecular mechanism of magnesium in reducing the adverse effects of PCOS phenotypes is not clearly understood. In general, magnesium is required for autophosphorylation of the tyrosine kinase receptor (insulin receptor). Phosphorylated tyrosine kinase is related to increased insulin sensitivity [21]. In addition, the activation of the tyrosine kinase receptor triggers the translocation of GLUT 4 to the plasma membrane; this leads to the increased uptake of glucose by the cell [22], resulting in the regulation of blood glucose concentrations. On the other hand, low intracellular magnesium causes decreased autophosphorylation of tyrosine kinase, which leads to IR. Women with PCOS also experience IR and low concentrations of magnesium that are similar to subjects with type 2 diabetes mellitus (T2DM). Low magnesium also triggers inflammation that, in turn, exacerbates IR [23]. Therefore, treating IR with magnesium supplementation offers an attractive strategy for patients with PCOS.

It is important to note that magnesium supplementation is associated with adverse symptoms of the gastrointestinal tract. These include nausea, vomiting, and osmotic diarrhea. Hypermagnesemia may also cause an irregular heartbeat, a decrease in lung function, decreased blood pressure, polydipsia, coma, and death. Patients with renal insufficiency or renal disease should use caution when taking magnesium supplements because magnesium excretion is severely limited in these patients; this may lead to hypermagnesemia, and the adverse effects of magnesium are more likely to occur [12]. Hypermagnesemia is likely to occur with a dose several times higher than the Upper Intake Level (UL). The tolerable UL of magnesium is 350 mg/d [24].

### 3.2. Zinc

Zinc is an essential trace mineral that plays a vital role in more than 100 enzymatic reactions and is part of about 3000 proteins. Zinc is predominately present inside the cell. Zinc biological functions include cofactor for enzymes (enzyme activation and inhibition), macronutrient metabolism, DNA and RNA metabolism (synthesis, repair, and replication), cell differentiation and growth, boosting immunity (anti-cancer defense and wound healing), regulation of signal transduction, and insulin and glucose metabolism. A plasma concentration of ˂60 µg/dL of zinc is considered deficient [25]. Zinc deficiency can occur with reduced intake (low meat consumption or vegetarianism/veganism), reduced absorption (due to increased fiber from plant-based foods and increased phytates from unleavened bread), increased losses, and increased demand (growth periods).

The role of zinc in reproduction has been known for some time. Specifically, zinc functions in gametogenesis (spermatogenesis and oogenesis) and fertilization [26]. The role of zinc in male reproduction has been well documented. On the other hand, the role of zinc in female reproduction is not clearly understood. Recently, the role of zinc in endocrine diseases such as PCOS has received some attention. Kanafchian et al. [27] conducted a case-control study in 60 women (20–40 years old) to study the serum zinc concentrations in women with PCOS. They reported a significantly low concentration of serum zinc in women with PCOS compared to controls (81.3 µg/dL vs. 108.3 µg/dL; *p* = 0.022). Several studies reported a positive impact of zinc supplementation on the metabolic and endocrine manifestations of PCOS [28,29,30]. These metabolic improvements associated with zinc supplementation can be attributed to the antioxidant properties exhibited by zinc [5,26,31].

Briefly, Foroozanfard et al. [28] conducted a randomized, placebo-controlled, double-blind study in women with PCOS (*n* = 52; 18–40 years old) to examine the effect of zinc supplementation on the metabolic profile of women with PCOS. Women were randomly divided into two groups. The treatment group received a supplement of 220 mg zinc sulfate (containing 50 mg elemental zinc) per day for 8 weeks. Compared to the placebo, zinc supplementation in women with PCOS resulted in a reduction in fasting plasma glucose (FPG) (−4.3 mg/dL, *p* = 0.03), serum insulin (−3.0 µIU/mL, *p* = 0.01), HOMA-IR (−0.8, *p* = 0.006), HOMA-IB (−10.6, *p* = 0.02), serum triglycerides (−15.6 mg/dL, *p* = 0.002), and very-low-density-lipoprotein (VLDL) cholesterol (−3.2 mg/dL, *p* = 0.002) and an increase in the quantitative insulin sensitivity check index [27]. Thus, zinc supplementation has been found to alleviate the metabolic (mostly MetSyn) pathologies associated with PCOS.

Zinc has been shown to exhibit anti-inflammatory and antioxidant properties. Jamilian et al. [29] conducted a randomized, placebo-controlled, double-blind study in women with PCOS (*n* = 48; 18–40 years old). Subjects were randomized into two groups of 24 subjects. One group received 220 mg of zinc sulfate supplementation (50 mg of elemental zinc), and another group received a placebo for 8 weeks. After 8 weeks, compared to placebo, zinc supplementation significantly reduced hirsutism (a reduction of 1.71 in Ferriman-Gallwey scores, a gold standard for the evaluation of hirsutism; *p* < 0.001) and decreased circulating malondialdehyde (MDA) concentrations (a marker of oxidative stress) (−0.09 µmol/L, *p* = 0.04). Although this study found a significant beneficial effect of zinc supplementation on alopecia, hirsutism, and plasma MDA, no effect was observed on the hormonal profiles of women with PCOS [29].

### 3.3. Selenium

Selenium is an essential trace mineral that is required in numerous biological metabolic functions, including carbohydrate and fat metabolism [32]. It also exhibits remarkable anti-inflammatory and antioxidant properties [33]. A randomized, double-blind, placebo-controlled study was conducted among 66 women with PCOS, aged 18–45 years, who were randomly treated with either 200 μg/d selenium or a placebo for 12 weeks. The objective of the research was to explore the impact of selenium supplementation on asymmetric dimethylarginine (ADMA), cardiometabolic risk factors, and hormonal status in women with PCOS. The results showed that selenium supplementation for 12 weeks had beneficial effects on the reduction of circulating ADMA and total testosterone concentrations in women with PCOS, without showing any correlation with hormone concentrations or lipid profile [34]. This finding was confirmed by another study conducted on 64 women (aged 18–40 years old) with PCOS who were administered 200 μg/d of selenium for 8 weeks. Significant decreases in reproductive outcomes, hirsutism, serum DHEA, hs-CRP, and MDA were reported [35].

On the other hand, Jamilian et al. [36] conducted a study in women with PCOS aged 18–40 years old (*n* = 70). Participants were randomly divided into two groups to receive either 200 μg/d of selenium supplements or a placebo for 8 weeks. Results showed a significant decrease in serum insulin concentrations (−29.8 pmol/L; *p* = 0.013), HOMA-IR (−1.15, *p* = 0.011), HOMA-B (−19.1, *p* = 0.017), serum triglycerides (−0.14 mmol/L, *p* = 0.025), and VLDL-cholesterol (−0.03 mmol/L, *p* = 0.025). The authors also reported a significant increase in the quantitative insulin sensitivity check index (+0.03, *p* = 0.032) [36]. Hajizadeh-Sharafabad et al. [32] conducted a systematic review of seven human studies. Only one out of seven studies that evaluated the effect of selenium supplementation on IR showed an antioxidant effect of selenium. Only two studies reported a VLDL and LDL cholesterol-lowering effect of selenium. The effect of selenium supplementation on androgens was inconsistent in subjects with PCOS [32]. In contrast, a randomized, placebo-controlled, double-blind study on 53 subjects with PCOS (18–42 years old) for 12 weeks revealed that selenium supplementation at a 200 µg dosage level significantly increased the IR compared to the placebo (2.05 vs. 1.81) [37]. Based on the recent evidence, the role of selenium supplementation in improving the metabolic pathologies associated with PCOS is inconclusive. Therefore, due to its toxicity, caution should be exercised in recommending large doses of selenium for PCOS [38].

The effect of the co-supplementation of selenium with probiotics has also been studied [39]. A double-blind, placebo-controlled clinical trial was conducted in 60 women with PCOS aged 18–40 years old. Participants were randomly divided into two groups and were treated with 8 × 10^9^ CFU/d probiotic plus 200 μg/d selenium or with a placebo for 12 weeks. After 12 weeks, the results revealed that women with PCOS who were treated with the co-supplement had a significant reduction in total testosterone, CRP, and MDA concentrations and improvements in hirsutism. In addition, there was a significant increase in the TAC. These beneficial effects can be attributed to the anti-inflammatory and antioxidant properties of probiotics and selenium [35,39].

### 3.4. Chromium

Chromium is an essential trace mineral. It plays a vital role in carbohydrate, protein, and lipid metabolism, as well as glucose and insulin homeostasis [40]. A systematic review and meta-analysis conducted by Fazelian et al. [41] reported that chromium supplementation had a beneficial effect on BMI (effect size, −2.37 kg/m^2^; 95% CI, −2.99, −1.76; *p* = 0.001), the concentration of free testosterone (effect size, −0.52 pg/mL; 95% CI, −0.83, −0.23; *p* = 0.001), and fasting insulin concentration (effect size, −0.86 mIU/mL; 95% CI, −0.67, −0.17; *p* = 0.001) in subjects with PCOS. A randomized, controlled study conducted on 54 women with infertile PCOS aged 18–40 years old showed a significant decrease in the concentrations of FPG, insulin, serum triglycerides, total cholesterol, and MDA, and a significant increase in plasma TAC [42].

A systematic review and meta-analysis of 28 RCT provided evidence for chromium supplementation for improved short-term (decreased fasting blood glucose concentrations; *p* < 0.001) and long-term glycemic control (decreased HbA1C concentrations; *p* = 0.004), decreased insulin resistance (decreased HOMA-IR; *p* < 0.001), and decreased insulin concentrations (*p* < 0.001) in T2DM subjects compared to the control group. [43]. Some of the metabolic features of T2DM are similar to PCOS. A systematic review of six studies showed no effect of chromium supplementation on weight reduction, glucose control, lipid profile, or hormonal disturbance for women with PCOS [44]. Similarly, Jamilian et al. [45] in a randomized, placebo-controlled, double-blind study reported a significant decline in fasting blood glucose (−2.3 mg/dL vs. 0.9 mg/dL, *p* = 0.03), insulin (−1.4 µIU/mL vs. 0.4 µIU/mL, *p* = 0.004), IR (−0.3 vs. 0.1, *p* = 0.005), serum triglycerides (−19.2 mg/dL vs. 8.3 mg/dL, *p* = 0.004), and total cholesterol (−15.3 mg/dL vs. −0.6 mg/dL, *p* = 0.03) in women with PCOS (18–40 years old) who received 200 µg/d chromium supplementation (*n* = 20) compared to those who received a placebo (*n* = 20). In addition, they found a significant increase in plasma TAC and MDA concentrations with chromium supplementation compared to placebo treatment [45]. In contrast, similar metabolic benefits were not observed in normoglycemic, non-obese subjects when chromium was supplemented at a 1000 µg dosage level for 16 weeks [46].

It has been known for some time that chromium improves glucose tolerance by reducing insulin resistance [47]. In a dose-dependent manner, chromium supplementation reduced circulating insulin, glucose, lipids, and glycated hemoglobin in patients with T2DM [48]. In addition, chromium increases pancreatic β-cell and insulin sensitivity, thereby improving insulin binding to its receptors and upregulating insulin receptor synthesis [49]. However, the recent evidence on the role of chromium in improving metabolic and clinical pathologies associated with PCOS is inconclusive. Caution needs to be exercised in recommending chromium supplementation because it is linked to oxidative stress, DNA damage, genomic instability, and carcinogenicity [50,51].

## 4. Conclusions

A brief summary of the findings on the role of magnesium, zinc, selenium, and chromium supplementation from selected studies is given in Table 1. In general, the analysis of the studies on the impact of magnesium, zinc, selenium, and chromium supplementation on various metabolic, clinical, and endocrine manifestations of PCOS provides a promising approach for medical nutrition therapy for PCOS. Supplementing patient diets with these micronutrients appeared to be beneficial in maintaining glucose and insulin homeostasis, improving the lipid profile, and increasing anti-oxidative capacity in women with PCOS. However, limited data are available on the role of minerals in hyperandrogenism and hirsutism in women with PCOS. Although increased adiposity, glucose intolerance, hyperinsulinemia, and insulin resistance (MetSyn pathologies) are common in women with PCOS, not all women exhibit these metabolic abnormalities [3]. Therefore, because of potential health risks, caution is needed in recommending these mineral supplementations (especially mega doses) to women with PCOS but without insulin resistance, glucose intolerance, and adiposity. Overall, minerals such as magnesium, zinc, selenium, and chromium can counteract the effects of oxidative and inflammatory stress associated with PCOS; therefore, supplementation with these minerals may ameliorate the pathologies of PCOS. In general, these minerals can also have a positive impact on other maladies at recommended doses. Due to higher bioavailability, an organic mineral salt such as gluconate, citrate, or aspartate may be a better choice for supplementation [12]. There is a need for further research to identify a specific type of mineral and dosage of that specific mineral for reducing the symptoms associated with PCOS. The toxicity of individual minerals needs to be taken into account before recommending a specific mineral or a combination of minerals for PCOS. Last but not least, persons with PCOS should be recommended to consume mineral-rich foods such as whole-grain cereals, nuts, seeds, legumes, leafy vegetables, and fruits. Toxicity is less likely to occur with foods as compared with supplement consumption.

## Figures and Tables

**Table 1:** Summary of studies that assessed supplementation of magnesium, zinc, selenium, and chromium in women with PCOS phenotype ^1^.

Reference (Author/s and Year)	Study Design	Mineral, Dosage, Duration, and Intervention	Outcome Measurement	Findings	Conclusions
[16]	Randomized, placebo-controlled, double-blind study	250 mg/d Mg oxide 8 wk *n* = 60	BMI WC Glycemic markers Lipid profile Androgens	↑BMI ↑WC No change in glycemic or lipid markers ↓Testosterone	Mg supplementation decreased BMI and androgens. Mg supplementation did not have an effect on glycemic and lipid markers
[17]	Randomized, placebo-controlled, double-blind study	250 mg/d Mg oxide &220 mg zinc sulfate (50 mg zn) 12 wk *n* = 60	hs-CRP TAC Gene expression	↓hs-CRP ↓Gene expression of TNF-α and IL-1 ↓TAC	Mg and Zn co-supplementation improved TAC and reduced inflammation
[28]	Randomized, placebo-controlled, double-blind study	220 mg/d Zn sulfate (50 mg Zn) 8 wk *n* = 52 Age 18–40 y	FBG Serum insulin HOMA-IR HOMA-B Lipid profile	↓FBG ↓Serum insulin ↓HOMA-IR and HOMA-B ↓Serum triglycerides ↓VLDL cholesterol	Zn supplementation improved several metabolic markers
[29]	Randomized, placebo-controlled, double-blind study	220 mg/d Zn sulfate (50 mg Zn) 8 wk *n* = 48 Age 18–40 y	Alopecia Hirsutism Malondialdehyde hs-CRP Androgens Cytokines	↓Alopecia ↓Hirsutism ↓Malondialdehyde No effect on androgens or Cytokines	Zn supplementation improved clinical symptoms and TAC
[34]	Randomized, placebo-controlled, double-blind study	220 µg/d Se 12 wk *n* = 66 Age 18–45 y	ADMA Glycemic profile Lipid profile Testosterone SHBG	↓Testosterone ↓Apo-B100/Apo-A1 ↓ADMA No effect on serum lipids, FBG, or SHBG	Se supplementation decreased androgens and had no effect on lipid profile
[36]	Randomized, placebo-controlled, double-blind study	200 µg/d Se 8 wk *n* = 70 Age 18–40 y	Serum insulin FBG HOMA-IR HOMA-B Lipid profile	↓Serum insulin ↓HOMA-IR ↓HOMA-B ↑Insulin sensitivity ↓Serum triglycerides ↓VLDL cholesterol	Se supplementation improved insulin sensitivity and lowered serum lipids but had no effect on FBG
[45]	Randomized, placebo-controlled, double-blind study	200 µg/d Cr 8 wk *n* = 40 Age 18–40 y	FBG Serum insulin HOMA-IR Lipid profile TAC Malondialdehyde	↓FBG ↓Serum insulin ↓HOMA-IR ↓Serum triglycerides ↓Total cholesterol ↓TAC ↓Malondialdehyde	Cr supplementation had a beneficial impact on glycemic control and lipid profile in infertile PCOS women

^1^ Abbreviations: ADMA, asymmetric dimethylarginine; BMI, body mass index; Cr, chromium; FBG, fasting plasma blood glues; HOMA-B; homeostatic model assessment for β-cell function; HOMA-IR, homeostatic model assessment for insulin-resistant; hs-CRP, high-sensitivity C-reactive protein; IL-1, interleukin-1; Mg, magnesium; PCOS, polycystic ovarian syndrome; Se, selenium; SHBG, sex hormone-binding globulin; TAC, total antioxidant capacity; TNF-α, tumor necrosis factor-α; VLDL, very-low-density-lipoprotein; WC, waist circumference; Zn, zinc. ^2^ ↑ represents a significant increase in the concentration of that biomarker; ↓ represents a significant decrease in the concentration of that biomarker.
